# Supplementary material for: Identification of photoperiod-induced specific miRNAs in the adrenal glands of Sunite sheep (Ovis aries)
Source: Front Vet Sci. 2022 Jul 22;9:888207. doi: 10.3389/fvets.2022.888207 (PMC9354845; doi:10.3389/fvets.2022.888207)
Supplement: Supplementary file 1 [file Data_Sheet_1.ZIP › Supplementary file/miRDeep2 file.zip!/miRDeep2 file/Novel_Structure/LP42A1/Novel_405.pdf]

Provisional ID : NC_019484.2_34954
Score total : 1.4
Score for star read(s) : -1.3
Score for read counts : 0
Score for mfe : 1.1
Score for randfold : 1.6
Score for cons. seed :
Total read count : 21
Mature read count : 21
Loop read count : 0
Star read count : 0

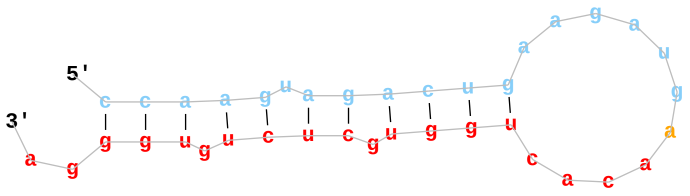

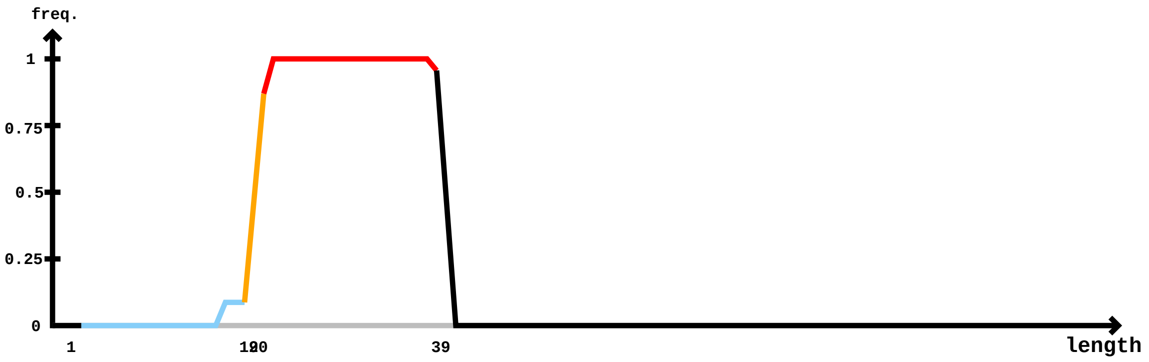

Star Mature

```
5'- cccaaguagacugaagaugaacacuggugcucugugggaugagguaguagauuguauaguuuuagggucauaccccaucuuggagauaacuauacagucuacugucuuu   -3'    exp
    ((((((.((((((...........)))).)))).))))..((((((((((((((((((((((((((((........))))))))....)))))))))))))))))))).   reads    mm    sample
    ................augaacacuggugcucuguggga......................................................................   2        0     seq
    ...................aacacuggugcucuguggga......................................................................   3        0     seq
    ....................acacuggugcucuguggga......................................................................   15       0     seq
    .....................cacuggugcucugugggg.......................................................................   1        0     seq
    .....................cacuggugcucuguggga......................................................................   2        0     seq
```
